# 2-Amino-5-(1*H*-tetra­zol-5-yl)pyridin-1-ium nitrate

**DOI:** 10.1107/S1600536808030791

**Published:** 2008-09-27

**Authors:** Jing Dai, Xiao-Chun Wen

**Affiliations:** a Ordered Matter Science Research Center, College of Chemistry and Chemical Engineering, Southeast University, Nanjing 210096, People’s Republic of China

## Abstract

In the cation of the title compound, C_6_H_7_N_6_^+^·NO_3_^−^, the pyridine and tetra­zole rings are essentially coplanar, exhibiting a dihedral angle of 6.30 (6)°. In the crystal structure, N—H⋯O, N—H⋯N, C—H⋯O and C—H⋯N hydrogen bonds form a three-dimensional network.

## Related literature

For general background on the chemistry of tetra­zole derivatives, see: Dunica *et al.* (1991 ▶); Wittenberger & Donner (1993 ▶); Zou *et al.* (2007 ▶); Xiong *et al.* (2002 ▶). For the crystal structures of related compounds, see: Dai & Fu (2008 ▶); Wang *et al.* (2005 ▶).
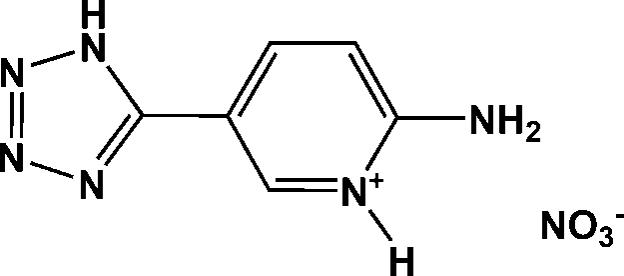


## Experimental

### 

#### Crystal data

C_6_H_7_N_6_^+^·NO_3_^−^
*M* *_r_* = 225.19
Monoclinic, 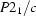

*a* = 8.3797 (17) Å
*b* = 6.9314 (14) Å
*c* = 15.881 (3) Å
β = 94.31 (3)°
*V* = 919.8 (3) Å^3^
*Z* = 4
Mo *K*α radiation
μ = 0.13 mm^−1^
*T* = 298 (2) K
0.30 × 0.22 × 0.20 mm

#### Data collection

Rigaku Mercury2 diffractometer
Absorption correction: multi-scan (*CrystalClear*; Rigaku, 2005 ▶) *T*_min_ = 0.916, *T*_max_ = 0.970
8766 measured reflections
2023 independent reflections
1520 reflections with *I* > 2σ(*I*)
*R*_int_ = 0.041

#### Refinement

*R*[*F* ^2^ > 2σ(*F* ^2^)] = 0.049
*wR*(*F* ^2^) = 0.120
*S* = 1.07
2023 reflections
173 parameters
All H-atom parameters refined
Δρ_max_ = 0.15 e Å^−3^
Δρ_min_ = −0.18 e Å^−3^

### 

Data collection: *CrystalClear* (Rigaku, 2005 ▶); cell refinement: *CrystalClear*; data reduction: *CrystalClear*; program(s) used to solve structure: *SHELXS97* (Sheldrick, 2008 ▶); program(s) used to refine structure: *SHELXL97* (Sheldrick, 2008 ▶); molecular graphics: *SHELXTL/PC* (Sheldrick, 2008 ▶); software used to prepare material for publication: *SHELXTL/PC*.

## Supplementary Material

Crystal structure: contains datablocks I, global. DOI: 10.1107/S1600536808030791/rz2247sup1.cif

Structure factors: contains datablocks I. DOI: 10.1107/S1600536808030791/rz2247Isup2.hkl

Additional supplementary materials:  crystallographic information; 3D view; checkCIF report

## Figures and Tables

**Table 1:** Hydrogen-bond geometry (Å, °)

*D*—H⋯*A*	*D*—H	H⋯*A*	*D*⋯*A*	*D*—H⋯*A*
N1—H1*A*⋯O2^i^	0.95 (2)	2.55 (2)	3.328 (2)	138.7 (18)
N1—H1*A*⋯O3^i^	0.95 (2)	1.84 (3)	2.764 (2)	163 (2)
N5—H5⋯O1^ii^	0.91 (2)	2.11 (2)	2.989 (2)	163 (2)
N5—H5⋯O3^ii^	0.91 (2)	2.21 (2)	2.908 (2)	133.3 (19)
N6—H6*A*⋯O1^iii^	0.88 (3)	2.31 (3)	3.074 (3)	145 (2)
N6—H6*B*⋯O2^iii^	0.90 (3)	2.48 (3)	2.908 (2)	110 (2)
N6—H6*B*⋯N2^iv^	0.90 (3)	2.32 (3)	3.176 (3)	158 (3)
C1—H1⋯O3^ii^	0.96 (2)	2.60 (2)	3.124 (2)	114.4 (16)
C1—H1⋯O2^i^	0.96 (2)	2.38 (2)	3.308 (2)	161.5 (18)
C3—H3⋯N4^v^	0.95 (2)	2.55 (2)	3.305 (3)	136.3 (16)

Symmetry codes: (i) 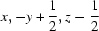; (ii) 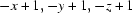; (iii) 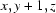; (iv) 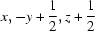; (v) 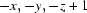.

**Fig. 1.**
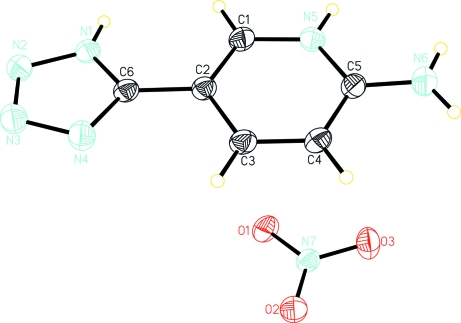
A view of the title compound with the atomic numbering scheme. Displacement ellipsoids were drawn at the 30% probability level.

**Fig. 2.**
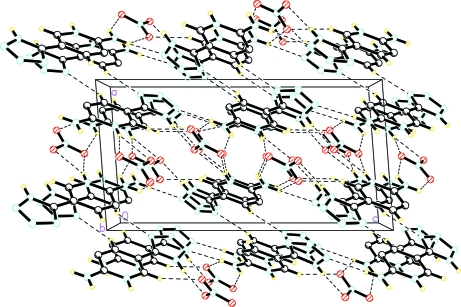
The crystal packing of the title compound viewed along the b axis showing the three-dimensionnal hydrogen bonding network (dashed lines). Hydrogen atoms not involved in hydrogen bonding are omitted for clarity.

## supplementary crystallographic information

### Comment

The tetrazole functional group has found a wide range of applications in coordination chemistry as ligand, in medicinal chemistry as a metabolically stable surrogate for the carboxylic acid group, and in materials science as high density energy material (Wang *et al.*, 2005; Xiong *et al.*, 2002; Zou *et al.*, 2007; Dunica *et al.*, 1991; Wittenberger & Donner, 1993). We report here the crystal structure of the title compound, 5-(1*H*-tetrazol-5-yl)pyridin-2-amine-1-ium nitrate.

In the cation of the title compound (Fig. 1) the pyridine and tetrazole rings are essentially coplanar with a dihedral angle of only 6.30 (6)°. Bond distances and angles of the tetrazole ring are within the usual range (Wang *et al.*, 2005; Dai & Fu, 2008). The pyridine N atom is protonated. The crystal packing is consolidated by N—H···O, N—H···N, C—H···O and C—H···N hydrogen bonds to form a three-dimentional network. (Table 1, Fig. 2).

### Experimental

2-Amino-5-cyanopyridine (30 mmol), NaN_3_ (45 mmol), NH_4_Cl (33 mmol) and DMF (50 ml) were added in a flask under nitrogen atmosphere and the mixture stirred at 110°C for 20 h. The resulting solution was then poured into ice-water (100 ml), and a white solid was obtained after adding nitrate acid (6 *M*) till pH=6. The precipitate was filtered and washed with distilled water. Colourless block-shaped crystals suitable for X-ray analysis were obtained from the crude product by slow evaporation of an ethanol/nitric acid (50:1 *v*/*v*) solution.

### Refinement

All H atoms were located in difference Fourier maps and refined freely.

### Crystal data

**Table:**

C_6_H_7_N_6_^+^·NO_3_^−^	*F*(000) = 464
*M**_r_* = 225.19	*D*_x_ = 1.626 Mg m^−^^3^
Monoclinic, *P*2_1_/*c*	Mo *K*α radiation, λ = 0.71073 Å
Hall symbol: -P 2ybc	Cell parameters from 1772 reflections
*a* = 8.3797 (17) Å	θ = 2.4–27.1°
*b* = 6.9314 (14) Å	µ = 0.13 mm^−^^1^
*c* = 15.881 (3) Å	*T* = 298 K
β = 94.31 (3)°	Block, colourless
*V* = 919.8 (3) Å^3^	0.30 × 0.22 × 0.20 mm
*Z* = 4	

### Data collection

**Table:**

Rigaku Mercury2 (2x2 bin mode) diffractometer	2023 independent reflections
Radiation source: fine-focus sealed tube	1520 reflections with *I* > 2σ(*I*)
graphite	*R*_int_ = 0.041
Detector resolution: 13.6612 pixels mm^-1^	θ_max_ = 27.1°, θ_min_ = 3.2°
ω scans	*h* = −10→10
Absorption correction: multi-scan (CrystalClear; Rigaku, 2005)	*k* = −8→8
*T*_min_ = 0.916, *T*_max_ = 0.970	*l* = −20→20
8766 measured reflections	

### Refinement

**Table:**

Refinement on *F*^2^	Primary atom site location: structure-invariant direct methods
Least-squares matrix: full	Secondary atom site location: difference Fourier map
*R*[*F*^2^ > 2σ(*F*^2^)] = 0.049	Hydrogen site location: inferred from neighbouring sites
*wR*(*F*^2^) = 0.120	All H-atom parameters refined
*S* = 1.07	*w* = 1/[σ^2^(*F*_o_^2^) + (0.0527*P*)^2^ + 0.2064*P*] where *P* = (*F*_o_^2^ + 2*F*_c_^2^)/3
2023 reflections	(Δ/σ)_max_ < 0.001
173 parameters	Δρ_max_ = 0.15 e Å^−^^3^
0 restraints	Δρ_min_ = −0.18 e Å^−^^3^

### Special details

**Table:**

Geometry. All e.s.d.'s (except the e.s.d. in the dihedral angle between two l.s. planes) are estimated using the full covariance matrix. The cell e.s.d.'s are taken into account individually in the estimation of e.s.d.'s in distances, angles and torsion angles; correlations between e.s.d.'s in cell parameters are only used when they are defined by crystal symmetry. An approximate (isotropic) treatment of cell e.s.d.'s is used for estimating e.s.d.'s involving l.s. planes.
Refinement. Refinement of *F*^2^ against ALL reflections. The weighted *R*-factor *wR* and goodness of fit *S* are based on *F*^2^, conventional *R*-factors *R* are based on *F*, with *F* set to zero for negative *F*^2^. The threshold expression of *F*^2^ > σ(*F*^2^) is used only for calculating *R*-factors(gt) *etc*. and is not relevant to the choice of reflections for refinement. *R*-factors based on *F*^2^ are statistically about twice as large as those based on *F*, and *R*- factors based on ALL data will be even larger.

### Fractional atomic coordinates and isotropic or equivalent isotropic displacement parameters (Å^2^)

**Table:**

	*x*	*y*	*z*	*U*_iso_*/*U*_eq_	
O1	0.49052 (18)	0.0891 (2)	0.58329 (9)	0.0552 (4)	
O2	0.3288 (2)	0.0420 (2)	0.68089 (9)	0.0632 (5)	
O3	0.46287 (18)	0.3054 (2)	0.67890 (10)	0.0588 (4)	
N7	0.42643 (19)	0.1424 (2)	0.64808 (10)	0.0406 (4)	
N1	0.2311 (2)	0.0535 (2)	0.27752 (10)	0.0416 (4)	
C2	0.2037 (2)	0.2485 (3)	0.40803 (11)	0.0359 (4)	
C5	0.2593 (2)	0.5628 (3)	0.51712 (11)	0.0401 (4)	
N5	0.3153 (2)	0.5556 (2)	0.43967 (10)	0.0427 (4)	
C4	0.1695 (2)	0.4035 (3)	0.54193 (12)	0.0411 (5)	
C6	0.1714 (2)	0.0831 (3)	0.35207 (11)	0.0367 (4)	
N4	0.0781 (2)	−0.0636 (3)	0.36762 (11)	0.0526 (5)	
N6	0.2911 (3)	0.7171 (3)	0.56527 (13)	0.0550 (5)	
C3	0.1443 (2)	0.2500 (3)	0.48954 (12)	0.0399 (4)	
N2	0.1724 (2)	−0.1144 (2)	0.24520 (10)	0.0497 (5)	
C1	0.2875 (2)	0.4049 (3)	0.38512 (12)	0.0414 (5)	
N3	0.0808 (2)	−0.1835 (3)	0.29971 (11)	0.0577 (5)	
H6A	0.345 (3)	0.815 (4)	0.5462 (15)	0.066 (8)*	
H6B	0.260 (4)	0.723 (4)	0.618 (2)	0.092 (10)*	
H1A	0.306 (3)	0.126 (3)	0.2484 (15)	0.066 (7)*	
H4	0.132 (2)	0.408 (3)	0.5949 (13)	0.045 (5)*	
H3	0.085 (3)	0.143 (3)	0.5074 (13)	0.052 (6)*	
H1	0.325 (3)	0.423 (3)	0.3298 (14)	0.056 (6)*	
H5	0.370 (3)	0.658 (3)	0.4211 (14)	0.061 (7)*	

### Atomic displacement parameters (Å^2^)

**Table:**

	*U*^11^	*U*^22^	*U*^33^	*U*^12^	*U*^13^	*U*^23^
O1	0.0669 (10)	0.0599 (10)	0.0416 (8)	0.0075 (8)	0.0238 (7)	−0.0058 (7)
O2	0.0879 (12)	0.0556 (9)	0.0497 (9)	−0.0239 (9)	0.0292 (9)	−0.0047 (7)
O3	0.0629 (10)	0.0531 (9)	0.0633 (10)	−0.0168 (8)	0.0236 (8)	−0.0186 (7)
N7	0.0451 (9)	0.0430 (9)	0.0345 (8)	0.0031 (7)	0.0084 (7)	−0.0002 (7)
N1	0.0519 (10)	0.0426 (9)	0.0314 (8)	−0.0025 (8)	0.0094 (7)	0.0003 (7)
C2	0.0373 (10)	0.0387 (10)	0.0321 (9)	−0.0009 (8)	0.0055 (7)	0.0016 (8)
C5	0.0445 (10)	0.0413 (11)	0.0344 (9)	0.0030 (8)	0.0015 (8)	−0.0005 (8)
N5	0.0499 (10)	0.0397 (9)	0.0391 (9)	−0.0064 (8)	0.0081 (8)	0.0028 (7)
C4	0.0457 (11)	0.0481 (11)	0.0302 (9)	−0.0006 (9)	0.0077 (8)	0.0010 (8)
C6	0.0395 (10)	0.0406 (10)	0.0306 (9)	−0.0012 (8)	0.0063 (8)	0.0029 (7)
N4	0.0623 (11)	0.0533 (11)	0.0440 (10)	−0.0183 (9)	0.0154 (9)	−0.0049 (8)
N6	0.0739 (14)	0.0439 (11)	0.0475 (11)	−0.0070 (10)	0.0060 (10)	−0.0057 (9)
C3	0.0414 (10)	0.0445 (11)	0.0344 (10)	−0.0052 (9)	0.0076 (8)	0.0037 (8)
N2	0.0650 (11)	0.0454 (10)	0.0392 (9)	−0.0052 (9)	0.0077 (8)	−0.0030 (8)
C1	0.0469 (11)	0.0453 (11)	0.0331 (10)	−0.0040 (9)	0.0101 (8)	0.0021 (8)
N3	0.0750 (13)	0.0515 (11)	0.0475 (10)	−0.0159 (10)	0.0118 (9)	−0.0056 (8)

### Geometric parameters (Å, °)

**Table:**

O1—N7	1.2517 (19)	N5—C1	1.366 (2)
O2—N7	1.221 (2)	N5—H5	0.91 (2)
O3—N7	1.260 (2)	C4—C3	1.358 (3)
N1—C6	1.336 (2)	C4—H4	0.92 (2)
N1—N2	1.350 (2)	C6—N4	1.317 (2)
N1—H1A	0.95 (2)	N4—N3	1.363 (2)
C2—C1	1.356 (3)	N6—H6A	0.88 (3)
C2—C3	1.422 (2)	N6—H6B	0.90 (3)
C2—C6	1.463 (3)	C3—H3	0.95 (2)
C5—N6	1.330 (3)	N2—N3	1.292 (2)
C5—N5	1.350 (2)	C1—H1	0.96 (2)
C5—C4	1.409 (3)		
			
O2—N7—O1	121.71 (17)	C5—C4—H4	117.3 (12)
O2—N7—O3	119.76 (16)	N4—C6—N1	108.35 (17)
O1—N7—O3	118.53 (16)	N4—C6—C2	125.21 (16)
C6—N1—N2	108.63 (16)	N1—C6—C2	126.44 (17)
C6—N1—H1A	131.0 (14)	C6—N4—N3	106.09 (16)
N2—N1—H1A	120.3 (14)	C5—N6—H6A	120.5 (16)
C1—C2—C3	117.54 (18)	C5—N6—H6B	120.9 (19)
C1—C2—C6	122.64 (16)	H6A—N6—H6B	119 (2)
C3—C2—C6	119.82 (17)	C4—C3—C2	120.98 (18)
N6—C5—N5	119.03 (19)	C4—C3—H3	119.4 (13)
N6—C5—C4	123.88 (19)	C2—C3—H3	119.6 (13)
N5—C5—C4	117.09 (17)	N3—N2—N1	106.44 (16)
C5—N5—C1	123.48 (17)	C2—C1—N5	120.56 (17)
C5—N5—H5	119.1 (15)	C2—C1—H1	123.9 (13)
C1—N5—H5	117.4 (15)	N5—C1—H1	115.4 (13)
C3—C4—C5	120.32 (18)	N2—N3—N4	110.49 (17)
C3—C4—H4	122.4 (13)		
			
N6—C5—N5—C1	−179.14 (19)	C2—C6—N4—N3	179.73 (19)
C4—C5—N5—C1	0.9 (3)	C5—C4—C3—C2	−1.7 (3)
N6—C5—C4—C3	−179.0 (2)	C1—C2—C3—C4	0.6 (3)
N5—C5—C4—C3	1.0 (3)	C6—C2—C3—C4	−178.50 (18)
N2—N1—C6—N4	0.7 (2)	C6—N1—N2—N3	−0.4 (2)
N2—N1—C6—C2	−179.74 (18)	C3—C2—C1—N5	1.2 (3)
C1—C2—C6—N4	−173.07 (19)	C6—C2—C1—N5	−179.71 (18)
C3—C2—C6—N4	6.0 (3)	C5—N5—C1—C2	−2.0 (3)
C1—C2—C6—N1	7.5 (3)	N1—N2—N3—N4	0.0 (2)
C3—C2—C6—N1	−173.47 (18)	C6—N4—N3—N2	0.5 (2)
N1—C6—N4—N3	−0.7 (2)		

### Hydrogen-bond geometry (Å, °)

**Table:**

*D*—H···*A*	*D*—H	H···*A*	*D*···*A*	*D*—H···*A*
N1—H1A···O2^i^	0.95 (2)	2.55 (2)	3.328 (2)	138.7 (18)
N1—H1A···O3^i^	0.95 (2)	1.84 (3)	2.764 (2)	163 (2)
N5—H5···O1^ii^	0.91 (2)	2.11 (2)	2.989 (2)	163 (2)
N5—H5···O3^ii^	0.91 (2)	2.21 (2)	2.908 (2)	133.3 (19)
N6—H6A···O1^iii^	0.88 (3)	2.31 (3)	3.074 (3)	145 (2)
N6—H6B···O2^iii^	0.90 (3)	2.48 (3)	2.908 (2)	110 (2)
N6—H6B···N2^iv^	0.90 (3)	2.32 (3)	3.176 (3)	158 (3)
C1—H1···O3^ii^	0.96 (2)	2.60 (2)	3.124 (2)	114.4 (16)
C1—H1···O2^i^	0.96 (2)	2.38 (2)	3.308 (2)	161.5 (18)
C3—H3···N4^v^	0.95 (2)	2.55 (2)	3.305 (3)	136.3 (16)

Symmetry codes: (i) *x*, −*y*+1/2, *z*−1/2; (ii) −*x*+1, −*y*+1, −*z*+1; (iii) *x*, *y*+1, *z*; (iv) *x*, −*y*+1/2, *z*+1/2; (v) −*x*, −*y*, −*z*+1.
